# Emerging Trends in Ultrasonic and Friction Stir Spot Welding of Polymers and Metal-Polymer Hybrids: A Review of Process Mechanics, Microstructure, and Joint Performance

**DOI:** 10.3390/ma19081602

**Published:** 2026-04-16

**Authors:** Kanchan Kumari, Swastik Pradhan, Chitrasen Samantra, Manisha Priyadarshini, Abhishek Barua, Debabrata Dhupal

**Affiliations:** 1 Department of Mechanical Engineering, Parala Maharaja Engineering College, Berhampur 761003, Odisha, India; kanchan.1087@gmail.com (K.K.); chitramech4u@gmail.com (C.S.); 2 School of Mechanical Engineering, Lovely Professional University, Phagwara 144411, Punjab, India; swastik.rock002@gmail.com; 3 CGU BEST Centre, C V Raman Global University, Bhubaneswar 752054, Odisha, India; any.manisha@yahoo.com.au; 4 Department of Automobile Engineering, Parala Maharaja Engineering College, Berhampur 761003, Odisha, India; 5 Department of Production Engineering, Veer Surendra Sai University of Technology, Burla 768018, Odisha, India; ddhupal_pe@vssut.ac.in

**Keywords:** friction stir spot welding, polymer joining, metal-polymer hybrids, mechanical characterization, numerical modeling, ultrasonic welding

## Abstract

The growing need for lightweight, multifunctional, and high-performance structures in the automotive, aerospace, electronics, and medical industries has driven the development of advanced joining technologies for polymers and metal-polymer combinations. Among these, ultrasonic welding (USW) and friction stir spot welding (FSSW) have emerged as promising solid-state techniques capable of producing reliable joints with minimal thermal degradation and enhanced interfacial bonding. This review focuses on recent developments in USW and FSSW of thermoplastics, fiber-reinforced composites, and hybrid metal–polymer systems, with a particular emphasis on process mechanics, microstructural evolution, and joint performance. The mechanisms of heat generation, material flow behavior, and consolidation are discussed in relation to key process parameters, including applied pressure, rotational speed, vibration amplitude, plunge depth, and dwell time. Microstructural transformations such as polymer chain orientation, recrystallization, interfacial diffusion, and defect formation are analyzed to establish process–structure–property relationships. Mechanical performance metrics, including lap shear strength, fatigue resistance, impact behavior, and environmental durability, are critically compared across different materials and welding methods. Furthermore, recent advances in numerical and thermo-mechanical modeling, in situ process monitoring, and data-driven optimization are discussed to highlight pathways toward predictive and scalable manufacturing. Current industrial applications and existing limitations such as challenges in automation, thickness constraints, and hybrid material compatibility are also evaluated. Finally, key research gaps and future directions are identified to improve joint reliability, sustainability, and broader industrial adoption of advanced solid-state welding technologies.

## 1. Introduction

The past few decades have seen an increase in the demand of lightweight, high-performance hybrid structures made of polymers and metals as a result of the requirement of improved specific strength, corrosion resistance, and design flexibility. These composite materials are the fusions of high-stiffness and load bearing characteristics of metals and low-density and manufacturing characteristics of polymers hence are suitable in the next generation structural components. Nonetheless, the incorporation of such different materials has been a major challenge because of their basic difference in thermal, mechanical and chemical attributes. As an illustration, polymers generally have low thermal conductivity and melting temperatures than metals, making it difficult to use industrial methods of joining (e.g., welding or brazing) without causing defects or weak interfaces. On this basis, some other frictional and solid-state joining techniques have been investigated further to provide dependable high-performance metal-polymer hybrid joints, including Ultrasonic Welding (USW) and Friction Stir Spot Welding (FSSW) [1].

**Figure 1:**
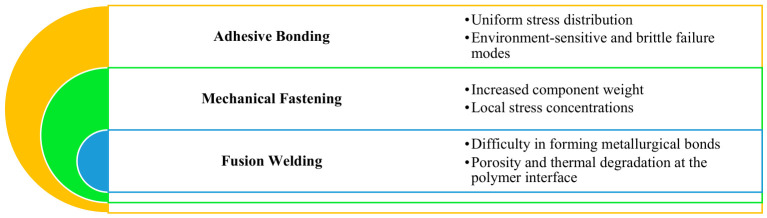
Limitations of conventional joining.

Traditional joining methods such as adhesive bonding, mechanical fastening, and fusion welding exhibit significant drawbacks when applied to metal–polymer assemblies (See Figure 1). Adhesive bonding can offer uniform stress distribution but is often sensitive to environmental conditions, exhibits poor long-term durability, and requires prolonged curing times. Mechanical fastening introduces additional weight and stress concentration points, reducing overall structural efficiency (See Table 1). Conventional hot or fusion welding techniques are challenged by the vastly different melting behaviors and bonding mechanisms of polymers and metals, often resulting in insufficient interfacial bonding, porosity, and residual stresses [2].

**Figure 2:**
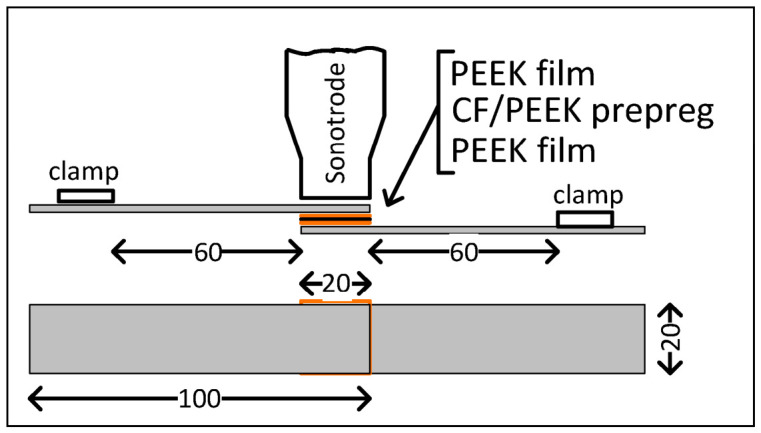
Schematic representation of the material stack for USW [4].

**Figure 3:**
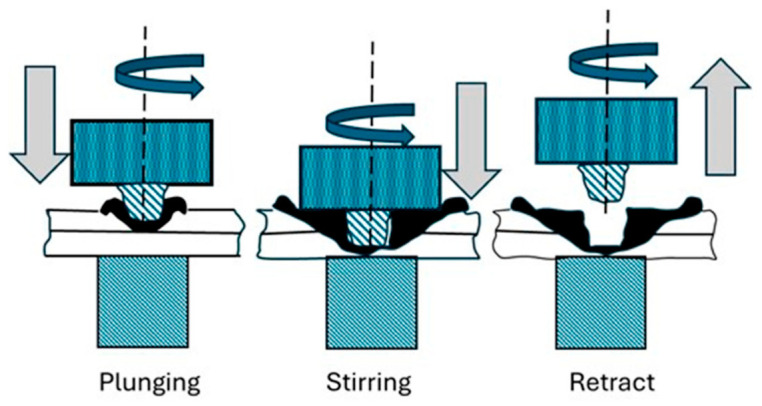
Stages of FSSW process [5].

Solid-state joining techniques such as USW and FSSW avoid complete melting of the base materials, relying instead on mechanical vibration (USW) or frictional heat and material flow (FSSW) to generate strong joints with reduced defects. USW applies high-frequency, low-amplitude vibrations at the interface between the joining partners, generating localized heat through friction and plastic deformation that results in a solid-state bond (see Figure 2). It is characterized by clean operation (no fumes or sparks), minimal surface damage, high production rates, and suitability for thin sections and complex geometries. However, its limitations include constraints on joint thickness and size due to limited vibrational energy penetration, as well as sensitivity to material stiffness and damping characteristics [3]. FSSW, a derivative of friction stir welding (FSW), employs a rotating tool to generate frictional heat and induce plasticized material flow at the tool–workpiece interface (see Figure 3). This process facilitates solid-state bonding, producing joints with a refined microstructure, reduced residual stresses, and improved mechanical reliability, particularly in metal–polymer hybrid systems. Since FSSW operates below the melting point of the materials, it minimizes common fusion welding defects such as cracking, porosity, and undesirable intermetallic formation [1].

This review presents an extensive and critical summary of the current trends in USW and FSSW of polymers and metal–polymer joints, addressing the major limitations of conventional joining technologies. Through a methodical integration of experimental studies and computational modeling analyses, the review clarifies the underlying mechanisms of process behavior, including heat generation and material flow patterns that govern joint formation in dissimilar material systems. A special focus is placed on the interfacial microstructure development, bonding mechanisms, polymer flow behavior, and the metallurgical and physicochemical interactions occurring at the interface that influence joint integrity. The review further includes a comparative evaluation of USW and FSSW for fabricating hybrid metal–polymer structures, along with a critical analysis of key process parameters and their interrelationships. In addition, it examines joint performance metrics, including mechanical strength, fatigue resistance, and long-term durability. Through this comprehensive and structured approach, the review aims to bridge existing knowledge gaps and provide practical insights for the design, optimization, and industrial application of solid-state joining techniques in advanced lightweight and hybrid material systems.

## 2. Materials for Ultrasonic and Friction Stir Spot Welding

During solid-state joining processes such as USW and FSSW, it is not merely material choice but the inherent material properties that govern the selection of joining methods and process parameters, which ultimately determine joint quality and mechanical performance, including microstructural evolution. The materials employed in these processes typically include thermoplastics and their composites, lightweight metals and alloys, and metal–polymer hybrid systems. Each material class presents distinct challenges and opportunities related to heat generation, material flow, bonding mechanisms, and interfacial compatibility, which must be carefully controlled through appropriate welding parameters.

### 2.1. Thermoplastics and Polymer Composites Used in USW and FSSW

Thermoplastic polymers have favorable processability for USW and FSSW due to their ability to soften and consolidate under frictional heat without bulk melting (See Table 2). Early work on friction stir welding of polymers identified common engineering thermoplastics such as polypropylene (PP), polycarbonate (PC), polyethylene (PE), and acrylonitrile butadiene styrene (ABS) as suitable candidates due to their ductility and thermal properties, which facilitate friction-induced deformation and joint formation [6].

Laboratory studies demonstrate that thermoplastics reinforced with fibers or fillers (e.g., glass-fiber, carbon fiber) exhibit enhanced stiffness, strength, and wear resistance when joined via FSSW compared to neat polymers. For instance, the FSSW of additive manufactured carbon fiber-reinforced nylon composites has produced sound joints with decent mechanical performance, indicating the viability of FSSW for polymer composites [7].

In USW, thermoplastics such as polyetherimide (PEI) and composite laminates have been joined effectively. Specialized probe designs such as in differential ultrasonic spot welding yield larger weld areas and improved joint integrity by physically displacing the polymer and enhancing intermolecular interdiffusion at the interface [8].

Key considerations for thermoplastics in USW/FSSW:

- Glass transition temperature (T_g_) and melting behavior: Affects heat generation and consolidation mechanisms.
- Viscosity and flow characteristics: Govern material flow and interfacial mixing during FSSW.
- Reinforcement type and content: Influence joint strength and failure modes.

### 2.2. Lightweight Metals and Alloys for Hybrid Joints

Metals and lightweight alloys form the metallic counterparts in metal–polymer joints via FSSW and, in some cases, USW with specialized tooling. Traditional applications of FSSW focused on metals such as aluminum alloys, magnesium alloys, and steel, which provide high specific strength and stiffness for structural assemblies [6]. For hybrid metal–polymer joints, soft metals such as aluminum (e.g., AA6082-T6) are often paired with polymers such as PA6, where the contrast in thermal and mechanical properties influences joint integrity (See Table 3). Recent innovations in metal surface texturing, such as mechanical grooving, generate meso-mechanical interlocking features that improve joint performance by enhancing physical anchorage with the polymer matrix [9].

### 2.3. Polymer-Metal Hybrid Material Combinations

**Figure 4:**
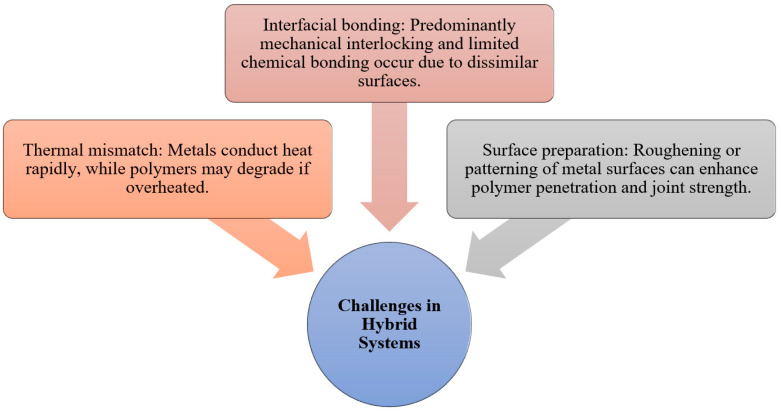
Different challenges in hybrid systems.

Joining dissimilar polymer–metal systems remains challenging due to significant differences in thermal conductivity, softening or melting behavior (for polymers), and rheological properties (see Figure 4). These disparities strongly influence heat generation, material flow, and interfacial bonding during solid-state joining. Systems such as polyethylene/AA5059 and PMMA/AA6061 have been investigated using friction-based solid-state welding techniques, demonstrating varying joint efficiencies and interfacial characteristics depending on processing parameters such as rotational speed, axial force, and welding time [1] (see Table 4). Previous studies also confirm that friction-based techniques can successfully join PMMA to metals and other polymers, where joint morphology and mechanical performance are governed by controlled welding conditions in both spot and linear configurations [10].

## 3. Fundamentals of Ultrasonic Welding

### 3.1. Working Principle and Process Stages

It is a solid-state joining process that utilizes high-frequency mechanical vibrations, typically in the range of 20–40 kHz, applied at the mating surfaces under controlled compressive pressure to generate localized heating and bonding at the interface. The welding system consists of an ultrasonic generator, transducer, booster, and sonotrode (horn), which together convert electrical energy into longitudinal mechanical vibrations that are transmitted to the joint interface. When polymer joining is being considered, the direction of vibration is usually perpendicular to the faying surfaces leading to oscillatory shear loads resulting in interfacial friction, viscoelastic heating and localized polymer melting [15]. The USW process is commonly described in three sequential stages:

- Surface adaptation and initial contact: The sonotrode applies a preset normal force, bringing the polymer components into intimate contact and conforming surface asperities to improve energy transmission [16].
- Vibration and energy input: Ultrasonic vibrations are introduced, leading to rapid heat generation at the interface due to intermolecular friction, hysteresis losses, and localized plastic deformation. This results in softening or melting of the polymer in a highly confined region [17].
- Consolidation and solidification: Once vibration ceases, the applied pressure is maintained during a brief hold time, allowing the molten or softened material to consolidate, interdiffuse, and solidify, thereby forming a weld upon cooling [18].

Owing to its extremely short cycle times, often less than 1–2 s, and the absence of external heat sources or filler materials, USW is particularly well suited for high-throughput manufacturing of thermoplastic components, offering precise energy control, minimal thermal distortion, and high repeatability [19].

### 3.2. Heat Generation Mechanisms and Viscoelastic Dissipation

Heat generation in the USW of polymers arises primarily from two coupled mechanisms: interfacial friction and viscoelastic dissipation within the polymer bulk (See Table 5). At the initial stage of vibration, microscopic asperities at the mating surfaces undergo relative oscillatory motion, generating localized frictional heating that raises the interfacial temperature. This process becomes dominant when significant softening is achieved. As the temperature approaches the T_g_, viscoelastic heating becomes the primary contributor to thermal energy generation. The thermoplastics also have viscoelastic behavior in time depending on cyclic loadings in which internal molecular friction transforms mechanical vibration to heat through a narrow subsurface layer. From a dynamic mechanical perspective, the rate of heat generation can be described using viscoelastic models under sinusoidal excitation. The volumetric heat generation rate (q, in W/m^3^) is commonly expressed as proportional to ω·E″·ε^2^, where ω (rad/s) is the angular frequency, E″ (Pa) is the loss modulus representing energy dissipation, and ε is the dynamic strain amplitude [20]. This relationship highlights that heat generation strongly depends on vibration amplitude, excitation frequency, and the intrinsic viscoelastic properties of the polymer, which govern material-specific responses during USW.

### 3.3. Role of Vibration Amplitude, Frequency, and Pressure

The three process parameters, which include amplitude of vibration, frequency, and the pressure of the static welding, determine the performance of the USW and have become interdependent. As a result, they control energy input and heat localization as well as interfacial consolidation (See Table 6).

- The amplitude of vibration at the interface is directly proportional to the level of cyclic strain (typically 10–100 µm). Increased amplitude enhances viscoelastic dissipation and melting rate; however, excessive amplitude may lead to non-uniform melting, flash formation, fiber damage (in composites), or thermal degradation [21].
- The frequency is usually designed to match the resonant frequency of the transducer-horn (usually 20, 30, or 40 kHz) to achieve the highest appeal to energy transfer efficiency. Although the customary difference in frequency is not feasible in practice, its impact on the depth of penetration, heating, and the treatment reaction of materials has been defined both analytically and numerically [22].
- Welding pressure (clamping force) provides direct intimate contact between mating surfaces providing an effective heat generation through friction and setting the thickness of the molten layer. Lack of pressure will result in poor contact and poor joints, but excessive pressure can force molten material out and lower joint integrity [23].

The experimental ones always indicate that the best of joint strength is reached with-in intermediate amplitude-pressure windows, which balance how much heat it generates and how well it controls the flow of the materials [24].

### 3.4. Energy Directors and Interfacial Morphology

Energy directors (EDs) are intentionally designed geometric features, commonly triangular, semicircular, or rectangular protrusions, introduced at the polymer interface to concentrate ultrasonic energy and accelerate local melting during the early stages of welding [25]. Due to their reduced cross-section, EDs experience elevated stress and viscoelastic heating, enabling rapid initiation of melt flow and intimate contact between polymer chains (See Table 7). ED geometry strongly influences melt distribution, void formation, and weld consistency, with triangular profiles often providing efficient energy focusing and controlled collapse behavior [26]. The resulting interfacial morphology, governed by ED shape, surface roughness, and joint design, controls polymer flow, interdiffusion, and consolidation. Properly engineered interfaces improve joint strength and promote mixed cohesive–adhesive failure modes, whereas suboptimal designs can lead to voids, incomplete fusion, or interfacial debonding [27]. In polymer–metal hybrid USW variants, analogous energy-directing effects are achieved through metallic surface texturing, coatings, or polymer interlayers, which promote localized melting, mechanical interlocking, and enhanced load transfer [28].

## 4. Fundamentals of Friction Stir Spot Welding

### 4.1. Process Principle and Tool-Workpiece Interaction

FSSW is a solid-state joining technique derived from linear friction stir welding, in which a rotating, non-consumable tool generates frictional heat to plastically soften the materials without reaching their melting point. In a typical lap joint configuration, the tool shoulder contacts the top surface while the pin penetrates into the layered sheets, producing localized heating and plastic deformation at the interface. This results in a metallurgical bond formed through intense stirring and forging action, thereby minimizing thermal distortion and defects associated with fusion welding [29].

**Figure 5:**
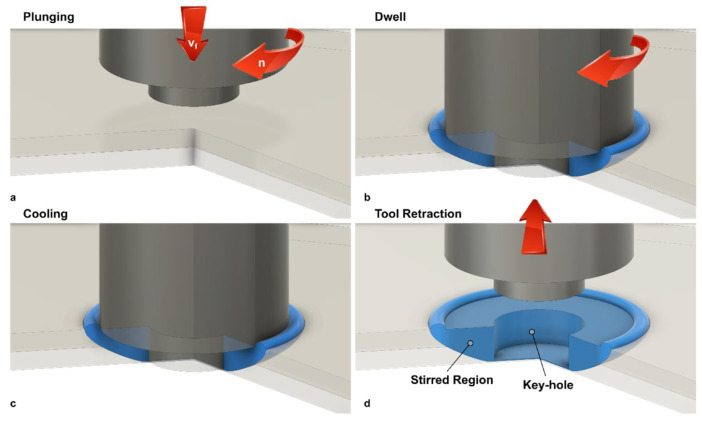
Schematic of FSSW showing different phases [31].

**Figure 6:**
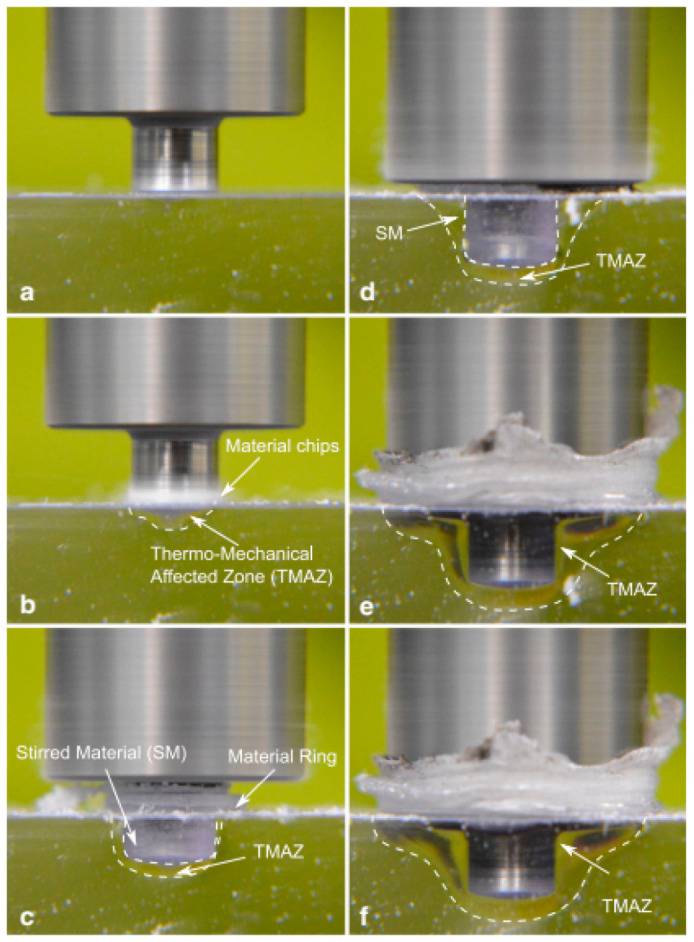
Schematic of FSSW showing different flow contours [31].

The sequential stages of the FSSW process, plunging, dwell, cooling, and retraction, are illustrated in Figure 5. During the plunging stage, the rotating tool penetrates the material, generating initial heat. In the dwell phase, the tool remains in position, allowing sufficient heat generation and material mixing. Cooling occurs upon tool withdrawal, followed by retraction, which leaves a consolidated joint. Joint quality in FSSW is governed by the interaction between the tool and the workpiece. The tool shoulder primarily controls heat generation and material confinement, while the pin facilitates stirring and intermixing within the weld zone. This combined action determines the weld nugget geometry and the extent of the stir zone. The resulting material flow behavior, including vortex formation and plasticized material movement, is depicted in Figure 6, which highlights the complex flow contours responsible for joint integrity and mechanical performance. Core actions in FSSW tool interaction are

- Frictional heating at tool shoulder/material contact elevates temperature to near but below melting [30].
- Plunge penetration forces material into the stirred zone, initiating solid-state bonding [31].
- Rotational shearing and plastic flow cause interfacial mixing and consolidation [30].
- Tool shoulder forging improves material density and interfacial contact [1].

### 4.2. Heat Generation and Material Flow Behavior

**Figure 7:**
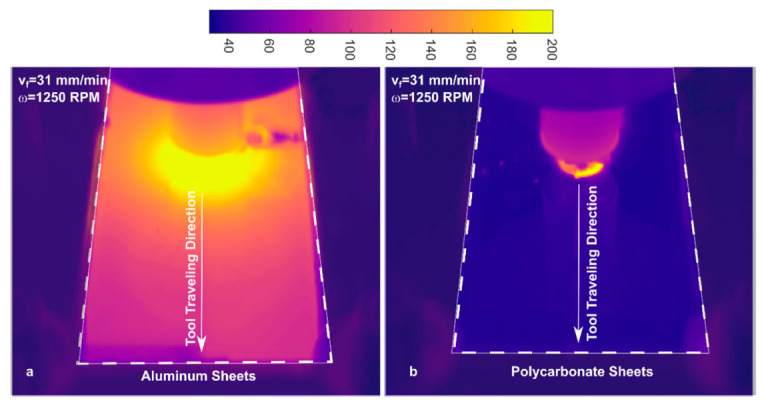
Thermal maps distribution in metals and polymers during FSSW [31].

The fundamental mechanisms of FSSW involve heat generation and material flow. Frictional heat arises from the interaction between the rotating tool (shoulder and pin) and the workpiece, along with plastic deformation within the stir zone. The majority of heat is generated at the tool shoulder due to its larger contact area, while the pin contributes to localized heating and material mixing. The thermal distribution during FSSW differs significantly between metals and polymers, as illustrated in Figure 7. In metals, high thermal conductivity enables rapid heat dissipation, resulting in a relatively broader softened zone around the tool. In contrast, polymers and polymer-based hybrids exhibit low thermal diffusivity, leading to steep thermal gradients and highly localized heating. When the temperature exceeds the T_g_, polymer chains gain mobility, significantly altering flow behavior compared to metallic systems. This distinction plays a critical role in determining joint formation mechanisms and final weld properties. Mechanisms in the flow of materials are triggered by

- Shear rotation caused by the pin profile;
- Development of thermo-mechanical affected zone (TMAZ) around the tool where the micro-structure is softened because of increased temperature;
- Following expansion of the stir region progressively with flow of heat and plastic flow.

Thermal gradients are typically more pronounced in polymers than in metals due to their lower thermal diffusivity, leading to localized heat accumulation. In terms of viscoplastic flow, polymers above their T_g_ exhibit deformation primarily through molecular chain rearrangement rather than dislocation motion as in metals. Additionally, geometry evolution during processing is governed by tool plunge depth and dwell time, which significantly influence the size, shape, and material reflow within the stir zone.

### 4.3. Tool Design, Plunge Depth, and Dwell Time Effects

Tool geometry, plunge depth, and dwell time critically influence heat generation, material flow, and overall joint quality in the process (see Table 8). Tool design particularly shoulder diameter, pin diameter, pin profile (cylindrical, threaded, conical), and surface features governs frictional heat distribution and material stirring; larger geometries enhance heat input and mixing but may also lead to defects such as flash if excessive. Plunge depth determines the extent of material plasticization and stir zone formation, where increased depth improves interfacial bonding but excessive penetration can cause material expulsion and structural weakening. Similarly, dwell time controls the duration of heat input and material consolidation; while longer dwell enhances weld strength and nugget formation, excessive exposure may degrade polymer components and reduce process efficiency.

### 4.4. Differences Between FSSW of Metals and Polymers

FSSW principles are quite comparable to those of metals and polymers, but significant differences occur since material thermal and rheological behavior differ:

- Thermal response metals are characterized by broad heat diffusion and relatively uniform softened regions, whereas polymers cannot diffuse, so heat accumulates in regions, forming steep thermal gradients (See Table 9).
- Plastic deformation: In metals, plastic deformation is mainly dislocation based, but in polymers, it is complex viscoelastic chain mobility and complex viscoplastic chain mobility above T_g_.
- Characteristics of the weld: Polymer FSSW can also produce high-quality stir zones with a substantial amount of the molecular mixing, and metallic FSSW can produce refined grains because of the dynamic recrystallization.
- Heat management: Tool design and parameters need to consider unique attributes of heat dissipation-attracting higher local control of polymers.

## 5. Process Mechanics and Material Flow

### 5.1. Deformation and Flow Behavior in Polymers

When joining polymers in solid state, it is important to study the deformation and flow behavior of materials as a thermoplastics consisting of polymers does not follow a thermos-product rheological behavior like a thermometal. In friction-based welding (FSSW) a spinning tool is used, which produces frictional and viscoelastic heat, raising the temperature of the polymer beyond its T_g_ and softening range, allowing it to flow without complete melting. During the FSSW of polymers, heat is also very concentrated around the tool due to the low thermal diffusivity of polymers; hence, the tool will have sharp temperature gradients and will have a small weld line, which is more likely to behave as a viscous fluid than as a solid. When the polymer is softened, the chains of the molecules become mobile and may mix together due to the rotation of the tool and the pressure posed by the forging, which leads to the flow of the material into the stir zone.

**Figure 8:**
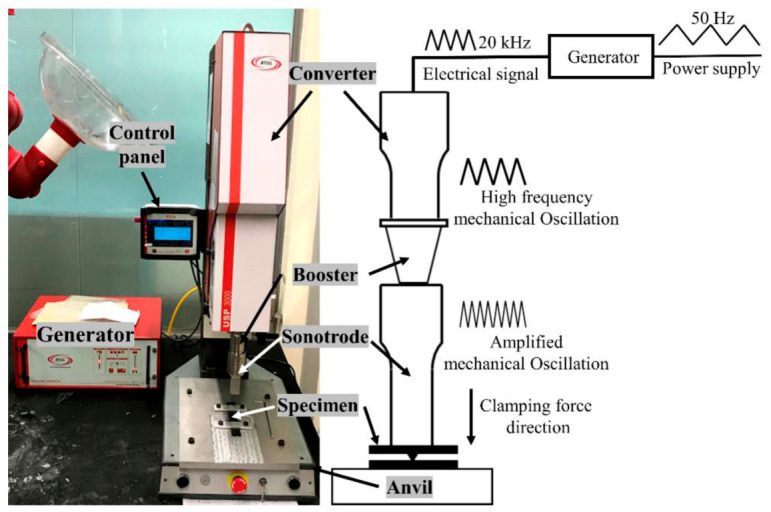
Schematic of the USW setup [19].

**Figure 9:**
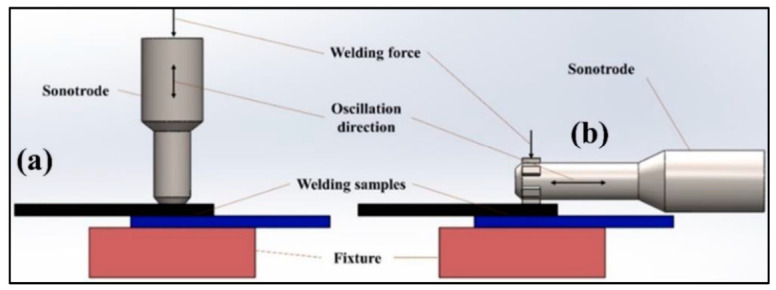
USW setup for (**a**) plastic, and (**b**) metal [19].

In USW of thermoplastics, mechanical vibrations at ultrasonic frequencies (~20–70 kHz) impart energy at the interface (see Figure 8). This induces viscoelastic heating and surface friction, rapidly softening the thermoplastic surfaces and causing localized melting and flow (See Figure 9). With a combination of static pressure and high-frequency oscillation, the softened polymer flows and fuses when cooling occurs under clamping force, enabling molecular interdiffusion and bond formation. USW thus relies on dynamic interface deformation rather than bulk flow [32]. Some key points are

- Polymer deformation during FSSW/USW is dominated by temperature-enabled flow of polymer chains rather than conventional plastic slip seen in metals.
- Lower thermal diffusivity in polymers leads to localized heat concentration and a narrow thermo-mechanically affected zone.
- In USW, viscoelastic and surface friction heat generation facilitates interfacial melting and polymer flow.

### 5.2. Plasticization, Stirring, and Consolidation Mechanisms

In FSSW, the process begins with a tool plunging into polymer sheets, generating frictional heat that progressively softens the thermoplastic near the tool. The rotating pin and shoulder then mechanically stir the softened material, distributing it around the weld nugget. The plasticization mechanism in polymers involves a combination of shear deformation and thermal softening, leading to enhanced flow and consolidation in the stirred zone. The softened polymer undergoes mechanical softening and pressure-induced flow, which fills voids and enhances interlayer adhesion. The flow of the materials in polymer-metal hybrids in FSSW is more complicated. The matrix polymer, which has been softened by frictional heat, then has the ability to press onto roughness or grooves on the surface of the partner metal aiding mechanical interlocking at the interface. Polymer flow under pressure and tool motion is directed to the interface as a result of pressure on the polymer, and the metal forms a firm backing, which once again results in macro-mechanical interlocking and micro-interdigitating mechanisms that help to form a strong joint. Mechanisms of material joining is

- Plasticization: Heat and deformation soften polymer chains.
- Stirring: Tool motion redistributes softened polymer into the weld region.
- Consolidation: Cooling under pressure fuses polymer chains and closes voids.
- Mechanical interlocking (hybrids): Polymer flows into metal roughness, aiding bond.

### 5.3. Interfacial Bonding Mechanisms in Metal–Polymer Joints

The principle bonding mechanism between the metal–polymer hybrid joints made by FSSW or other solid-state processes is inherently different to that of metal–metal joints (see Table 10). Having been heated beyond its softening point, the polymer is able to creep and flow to surface asperities and textures on the metallic partner. This provides a mechanical interlocking effect in which the polymer chains cover and fill the micro-features on the metal surface which provide improved load transfer and strength to the joints. In other instances, the adhesive interrelations between the polymer functional groups and the metal oxides also promote interfacial bonding particularly in the presence of surface pretreatments (e.g., grooving, etching with chemicals).

Not because the bond is chemically stronger, but because the polymer matrix mechanically attaches itself onto the surface features, experimental studies (e.g., grooved AA6082-PA6 joints) have demonstrated that texturing the metal surface before FSSW results in an improved mechanical performance [9].

USW is fast, highly localized, and is practical with small, thin sheets; though, it requires very close control of the extent of vibration and clamping pressure to achieve sufficient melting and consolidation of the material without causing the polymer to degrade. Conversely, FSSW is able to produce larger and stronger joints with a bulk material stirring, although the rotational speed, plunge depth, and dwell time must be optimized to allow control over heat input and to avoid such defects as voids, flash, and expelled material. The process of joining USW and FSSW in polymers and metal–polymer hybrids is fundamentally dissimilar to the process of metallic welding (See Table 11). Although the two processes are similar in the sense that the processes require the use of controlled heat generation and polymer softening as a prerequisite to bonding, the USW process is characterized by viscoelastic dissipation and interfacial melting which is followed by pressure-assisted consolidation, whereas the FSSW does the formation of the joint by heating of the material by friction and macroscopic flow of the material. In the hybrid system, joint integrity is majorly controlled by mechanical interlocking of the softened polymer into metallic surface features which is considerably boosted by surface texturing or pre-treatment. In-depth knowledge of these thermomechanical mechanisms with the help of microstructural characterization and physics-related modeling is thus a key to the further improvement of viable, high-performance joining of polymers and polymer-based hybrid structures in industries.

## 6. Microstructural Evolution in Welded Joints

The mechanical integrity and failure characteristics as well as the long-term behavior of the joint is regulated by microstructural changes during welding. The evolving microstructure under extreme deformation processes and thermal load during solid-state welding (FSSW and USW of polymer and metal–polymer hybrids) demonstrates different characteristics in polymer and metal and interfacial areas. The knowledge of these phenomena is essential in the process of optimizing process parameters and enhancing the joint strength, durability and reliability.

### 6.1. Polymer Chain Orientation and Recrystallization

In polymer welding, the orientation and crystallization of polymer chains has a high degree of impact in determining the performance of joints. USW generates localized high-frequency vibrations encapsulating molecular mobility and alignment of chains at the interface leading to enhancement of diffusion and interfacial entanglements, which is a major force in polymer–polymer systems that facilitates joint strength through time and temperature (e.g., chain entanglements enhance interfacial strength with time and temperature) [33]. Under thermal and mechanical action, partial recrystallization can occur in semi-crystalline polymers, refining microstructure and reducing anisotropy in mechanical response. Visualization of crystallinity changes can be achieved through differential scanning calorimetry (DSC) and X-ray diffraction (XRD), while molecular orientation effects are evident in birefringence and transmission electron microscopy (TEM) images. Some important facts are

- Ultrasonic vibrations enhance chain mobility and interfacial entanglement (See Table 12).
- Partial recrystallization refines polymer microstructure and improves stiffness/strength.
- Experimental evidence links enhanced interfacial strength to increased polymer chain interactions at bonded zones.

### 6.2. Metal Microstructural Changes, Interfacial Morphology, and Defect Formation in Metal–Polymer Welding

Metal–polymer welding processes such as USW and FSSW induce significant thermo-mechanical effects in the metallic region, leading to dynamic recrystallization (DRX), grain refinement, and the formation of distinct microstructural zones including the stir nugget, TMAZ, and HAZ. DRX within the stir zone produces fine, equiaxed grains with reduced dislocation density, typically enhancing hardness and strength relative to the base metal. Compared to conventional fusion welding, lower peak temperatures in these processes suppress excessive melting and limit the formation of brittle intermetallic compounds, thereby improving joint toughness and reliability; however, excessive heat input or prolonged dwell time may cause grain coarsening in the TMAZ, adversely affecting mechanical performance and highlighting the need for precise thermal control [34,35,36]. At the interface, joint strength is governed primarily by mechanical interlocking rather than metallurgical bonding, due to limited chemical affinity between metals and polymers. During welding, softened polymer flows into surface asperities, grooves, or textured features of the metal, forming interlocked regions observable in microstructural analysis. Surface pretreatments such as laser texturing, knurling, and micro-grooving significantly enhance interfacial adhesion by increasing contact area and promoting cohesive failure within the polymer (see Table 13), with interface morphology varying from sharp and rough to more gradual transitions depending on processing conditions [37]. Despite these advantages, defects remain a critical concern: voids and porosity may arise from polymer shrinkage or entrapped gases, reducing static and fatigue strength; excessive flash formation can either enhance interlocking or introduce surface irregularities; and “kissing bonds,” characterized by intimate but weakly bonded interfaces, are particularly detrimental to fatigue life. Additionally, the thermal degradation of polymers under excessive heat exposure can reduce molecular weight and mechanical properties, necessitating careful process optimization and characterization using techniques such as thermogravimetric analysis to define safe operating windows [38,39].

## 7. Mechanical and Functional Performance of Joints

### 7.1. Lap Shear Strength and Tensile Performance

Lap shear strength and tensile performance of hybrid joints depend on mechanical interlocking, interface quality, and process parameters. Mechanical grooving of metal surfaces for FSSW has been shown to create meso-mechanical interlocking, resulting in mixed adhesive/cohesive failure with improved joint strength compared to conventional grinding, which tended to yield adhesive failure modes (See Table 14). Quantitative strength results are as follows:

- Advanced seal-flow Friction Stir Lap Welding (FSLW) between aluminum and GF-PEEK achieved tensile shear strengths up to ~26 MPa, a ~28% increase over traditional FSLW joints, highlighting the importance of material flow and interlocking morphology on strength [40].
- Pre-treatment of surfaces before loading (e.g., phosphoric acid anodizing with primer) with polymer–metal friction spot joints indicated better maintenance of strength with age indicating that interface chemistry and adhesion have substantial contributions to the load-bearing capacity [41].

The balance between mechanical interlocking and the adhesive bonding in the interface is mainly a determining factor of the joint strength in polymer–metal hybrid welding. The importance of pretreatments and interfacial conditioning on the surface will be significant in reducing the loss of strength especially in environmental aging and when exposed to moisture. Also, the material flow behavior and joint configuration have a strong impact on lap shear strength and ultimate tensile performance of the joints due to their control of interfacial morphology, efficiency of load transfer, and failure mode.

### 7.2. Fatigue and Impact Behavior

(a) Fatigue Performance: Fatigue behavior is a major performance indicator for load-bearing joints (See Table 15). In metal–polymer friction spot joints, fatigue tests under cyclic loading (R = 0.1, 5 Hz) revealed that joints can sustain up to 10^6^ cycles at 25–35% of quasi-static strength without significant degradation in residual strength when later re-tested quasi-statically [42].
(b) Impact and Dynamic Loading: Polymer joints exhibit complex fatigue crack initiation and propagation associated with the polymer matrix, fiber orientation, and interface quality. For example, fatigue fracture surfaces show striations in resin-rich areas indicating mixed fatigue mechanisms within the polymer matrix and at the bonded interface [42].

The high resistance in the joints to the cyclic loading is usually accompanied by better intermixing of the materials in the interface, as well as effective surface treatments, which contribute to the enhanced transfer of the load and the delay of the crack initiation. Fatigue life often is found to be proportional to a fraction of the quasi-static strength, which further illustrates the paramount importance of interface durability and bonding integrity to long-run performance. Despite the relatively less attention that has been given to impact and dynamic loading behavior in the literature, they are vital in service conditions in terms of sudden or transient loads, which promotes a significant field to be pursued by experimental studies in the future.

### 7.3. Failure Modes, Fracture Mechanisms, and Environmental Durability

The microstructural characteristics developed during ultrasonically welded and friction-based polymer and metal–polymer hybrid joints play a decisive role in governing failure behavior. In particular, interfacial bonding and mechanical interlocking mechanisms dominate joint performance. The observed failure modes typically include adhesive failure at the interface, cohesive failure within the polymer matrix, or a mixed adhesive–cohesive mode. The behavior of adhesive failure is most common in joints that possess weak interfacial bonding, and those which have been subjected to environmental aging, and their moisture ingress reduces polymer–metal adhesion and encourages interfacial debonding [43].

**Figure 10:**
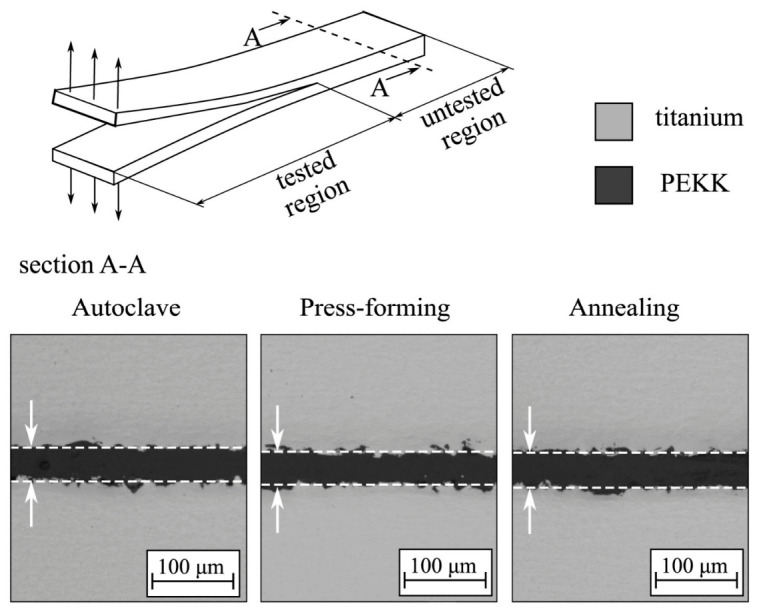
Schematic representation of cross-sectional microscopy images of metal–polymer joint [47].

Conversely, joints engineered with enhanced mechanical interlocking through surface texturing, grooving, or controlled material flow exhibit cohesive or mixed failure modes, indicating improved load transfer and structural integrity. These microstructural features, as illustrated in Figure 10, highlight variations in interfacial morphology under different processing routes such as autoclave forming, press forming, and annealing, thereby influencing bonding quality and failure characteristics.

Process parameters critically influence these outcomes. Variables such as rotational speed, dwell time, and heat input govern polymer softening, material flow, and interfacial consolidation during friction spot welding. Optimized conditions yield strong joints, with reported tensile strengths approaching ~70 MPa for PA6-based FSSW joints, whereas insufficient heat leads to incomplete bonding and excessive heat causes polymer degradation, flash formation, or material expulsion, ultimately reducing joint performance [44]. Environmental durability further affects long-term reliability. Accelerated aging studies report a 20–30% reduction in lap shear strength under prolonged humidity exposure. However, with appropriate surface pretreatments and interfacial engineering, joints can retain 50–80% of their original strength after extended environmental exposure. Moisture-induced degradation primarily weakens interfacial adhesion, promoting adhesive failure in poorly bonded systems [45,46]. Key observations include

- Replacement of adhesive failure to mixed or cohesive failure with enhanced mechanical interlocking and interfacial design.
- Close sensitivity of fracture behavior to process variables that determine heat input, material flow, and defect formation.
- Observable loss of strength during so-called environmental aging, and can be addressed substantially by improved surface treatment and interface engineering (See Table 16).

## 8. Numerical Modeling and Simulation Approaches

The numerical modeling is significant in the service of comprehending and optimizing the solid-state welding procedures of USW and FSSW. In comparison with purely experimental approaches, simulation can provide information on internal thermal fields, material movement, interfacial bonding, and defect formation, and thus allow for the prediction and optimization of joint quality without the need to run many trial-and-error experiments.

### 8.1. Thermal and Thermo-Mechanical Modeling of USW

Thermo-mechanical models are used to simulate heat generation and distribution during USW, which are critical for interfacial bonding and microstructural evolution. Heat is primarily generated through interfacial friction and viscoelastic dissipation at polymer–polymer or metal–polymer interfaces.

- Finite Element (FE) Methods: Coupled thermal–mechanical FE models are widely used to simulate frictional heat generation, transient heat transfer, and material deformation during welding.
- Viscoelastic and Contact Modeling: Temperature and strain-dependent viscoelastic constitutive models are applied to capture polymer softening and damping behavior under ultrasonic vibrations.
- Boundary Conditions and Contact Mechanics: Accurate definition of friction coefficients, thermal contact conductance, and vibration parameters is essential to predict realistic temperature and stress distributions in the weld zone.

### 8.2. CFD and Material Flow Modeling in FSSW

**Figure 11:**
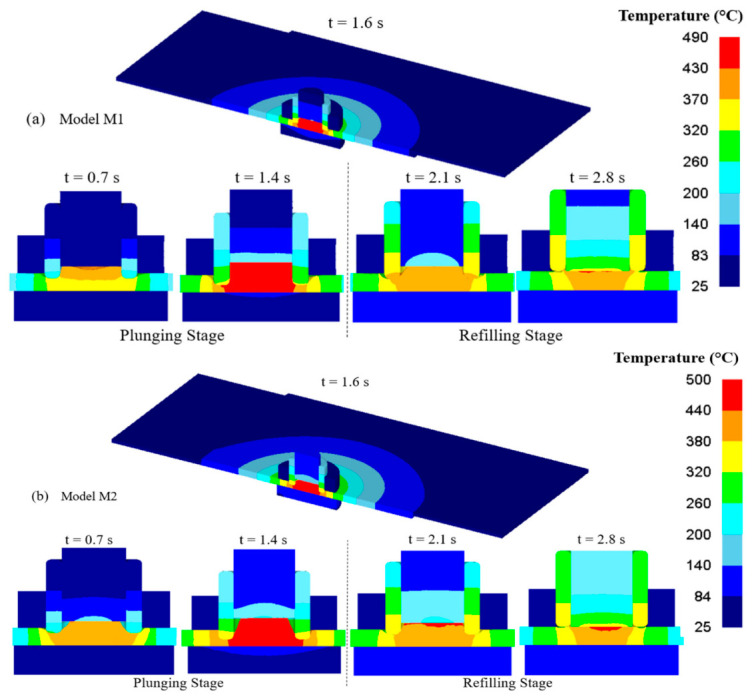
Schematic representation of FEA-based temperature distribution during the FSSW process obtained from simulations [50].

FSSW involves severe plastic deformation, complex contact conditions, and rapid material flow around the rotating tool. Numerical modeling therefore relies on both computational fluid dynamics (CFD) and thermo-mechanical finite element methods (Figure 11).

- CFD-Based Modeling: The plasticized material is often treated as a non-Newtonian viscous fluid. CFD models simulate shear stress, frictional heat generation, and momentum transfer to predict material flow patterns and defect formation such as voids [48].
- Thermo-Mechanical Coupled Models: Fully coupled FE approaches, particularly Coupled Eulerian-Lagrangian (CEL) formulations, are used to simultaneously solve heat transfer and material deformation. These models are effective for capturing large deformations without mesh distortion and are widely applied in FSSW and refill FSSW (RFSSW) [49].
- 3D Modeling and Process Parameter Effects: Three-dimensional simulations analyze the influence of tool rotation speed, plunge depth, and dwell time on temperature distribution, strain evolution, and material flow, supporting process optimization [50].

### 8.3. Constitutive Models for Polymers and Hybrids

Accurate simulation of USW and FSSW requires constitutive models that reflect the thermo-mechanical behavior of the materials involved:

- Johnson–Cook (J–C) Model: Often used for metals undergoing large plastic deformation, the J–C model accounts for strain hardening, strain-rate sensitivity, and thermal softening, making it suitable for metallic constituents in FSSW simulations. The Johnson–Cook Flow Stress Equation is expressed as [50]
  (1) $$\sigma =\left[A+B\varepsilon^{n}\right]\left[1+C\ln\left(\frac{\dot{\varepsilon }}{\dot{\varepsilon_{0}}}\right)\right]\left[1-{\left(\frac{T-T_{r}}{T_{m}-T_{r}}\right)}^{m}\right]$$
  where σ = Flow stress, ε = Equivalent plastic strain, $\dot{\varepsilon }$ = Plastic strain rate, $\dot{\varepsilon_{0}}$ = Reference strain rate, T = Current temperature, T_r_ = Room/reference temperature, T_m_ = Melting temperature, A = Yield stress at reference condition, B = Strain hardening coefficient, n = Strain hardening exponent, C = Strain rate sensitivity coefficient, m = Thermal softening exponent.
- Viscoelastic/Viscoplastic Models for Polymers: Polymer materials exhibit pronounced time-dependent deformation, strain-rate sensitivity, and significant thermal softening, particularly as the temperature approaches the T_g_. Unlike metals, their mechanical response is governed by molecular chain mobility, which results in coupled elastic, viscous, and plastic effects. Therefore, advanced simulation frameworks incorporate viscoelastic constitutive relations or temperature-dependent viscoplastic models to accurately represent polymer behavior under mechanical and thermal loading [51,52].
  (a) Viscoelastic Constitutive Modeling: For linear viscoelastic behavior, stress is expressed as a convolution integral:
    (2) $$\sigma \left(t\right)=\int_{0}^{t}G\left(t-\tau \right)\frac{d\varepsilon \left(\tau \right)}{d\tau }d\tau$$
    where *σ*(*t*) is the stress at the current time ‘*t*’, *ε*(*τ*) is the strain at past time ‘*τ*’, *G*(*t* − *τ*) is the relaxation modulus describing material response over time. This formulation is commonly implemented in finite element models using the generalized Maxwell (Prony series) representation:
    (3) $$G\left(t\right)=G_{\infty }+\sum_{i=1}^{n}G_{i}e^{-t/r_{i}}$$
    where $G_{\infty }$ = long-term modulus, G_i_ = relaxation modulus coefficients, $\tau_{i}$ = relaxation times.
  (b) Temperature Dependence (Time–Temperature Superposition): Near T_g_, viscoelastic response becomes strongly temperature sensitive. The Williams–Landel–Ferry (WLF) equation is widely used for construction of master curves for long-term behavior prediction.
    (4) $$\log a_{T}=\frac{-C_{1}\left(T-T_{r}\right)}{C_{2}+\left(T-T_{r}\right)}$$
    where $a_{T}$ = shift factor, ‘*T*’ is the absolute temperature at which the material response is evaluated, T_r_ = reference temperature (typically close to the T_g_), C1, C2 = material constants.
  (c) Viscoplastic (Rate-Dependent Plasticity) Modeling: When polymers deform beyond yield, irreversible flow occurs. A commonly used overstress-based model is the Perzyna viscoplastic formulation:
    (5) $$\dot{\varepsilon^{vp}}={\left(\frac{f\left(\sigma \right)}{K}\right)}^{m}$$
    where $\dot{\varepsilon^{vp}}$ = viscoplastic strain rate, $f\left(\sigma \right)$ = yield function, K = viscosity parameter, m = strain-rate sensitivity exponent.
    Temperature dependence of yield stress may be incorporated as
    (6) $$\sigma_{y}\left(T\right)=\sigma_{y^{0}}{\left(1-\frac{T}{T_{g}}\right)}^{n}$$
    which reflects the rapid softening of polymers near T_g_.
- Hybrid Material Models: For polymer–metal hybrids, constitutive formulations are often layered or multi-phase models that combine metallic plasticity with polymer viscoelasticity, enabling accurate representation of joint zones. Although the literature specifically on hybrid constitutive modeling for USW/FSSW is limited, emerging studies indicate the need for multi-scale and multi-physics frameworks that incorporate differences in thermal conductivity and modulus across the interface.

Table 17 contextualizes the selection of constitutive models for different material classes in USW and FSSW simulations, highlighting their relevance to thermo-mechanically coupled process modeling.

### 8.4. Model Validation with Experimental Results

**Figure 12:**
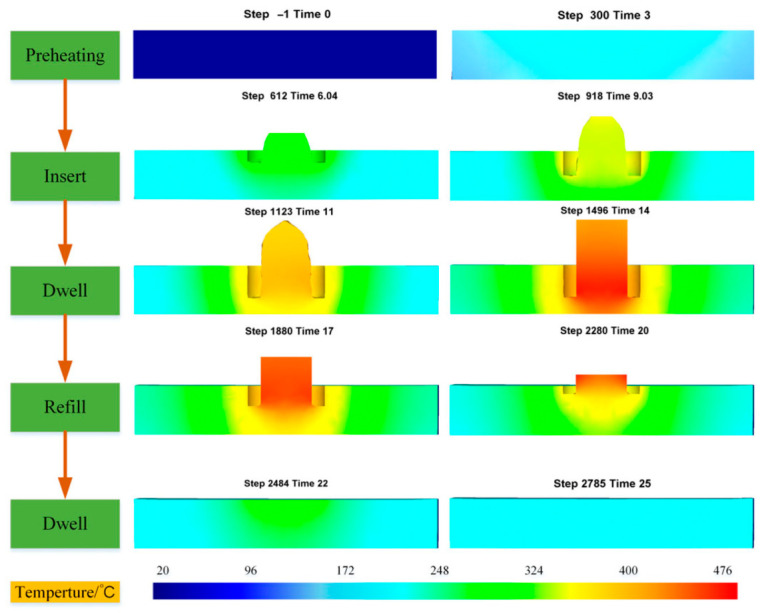
FEA-based temperature distribution showing temporal heat evolution and comparison of simulated contours with experimental thermocouple measurements in an FSSW lap joint [53].

Validation is a critical step that determines whether numerical predictions are reliable:

- Temperature Comparison: Thermocouple and infrared measurements are often used to validate simulated thermal fields (See Figure 12). Deviations within ±5% between simulation and experiment indicate robust models [53].
- Material Flow and Defect Prediction: Numerical models predict zones of high plastic strain and potential defects (voids/tunnels) which are then confirmed by microstructural examination and stop-action experimental snapshots [49].
- Joint Geometry and Macrostructure: Simulated weld cross-sections and nugget shapes are compared with actual joint macrographs to verify material flow patterns.

## 9. Process Optimization and Control Strategies

The application of optimization and control in the welding process is paramount to the repeatability of high-quality USW and FSSW in welding polymers and metal–polymer hybrids. The nonlinearity and coupling of the energy input, material flow, and the mechanisms of bonds between interfaces make the systematic form of optimization of such processes and real-time control strategies widely used as alternatives to the processes of parameter choice in the past. These approaches lie within the boundaries of the classical approaches to statistics, state-of-the-art machine learning and data-driven solutions, real-time surveillance, and energy efficiency solutions that are sustainability oriented.

### 9.1. Parameter Optimization Using DOE and Statistical Methods

The statistical experimental designs, particularly the Design of Experiments (DOE) methods like the Taguchi method and Response Surface Methodology (RSM), have continued to be the underpinning to the optimization of welding parameters. These methods facilitate the systematic exploration of issues of multi-factor interactions (rotational velocity, dwell time, amplitude, pressure, etc.) and reduce the number of runs in the experiments, while capturing the primary results on the strength and quality of joints. The example is that Taguchi optimization in USW of thermoplastic power case assemblies is that amplitude is the key variable of weld strength, then the weld pressure and hold time and confirmation experiments confirmed the optimum parameter set statistically [54]. Some Key Points are

- DOE consumes less effort in experiments and tests a number of parameters and interactions.
- ANOVA measures the importance and the contribution of all parameters.
- Multi-response optimization (desirability, RSM, grey relational analysis) is used to reproduce many of these performance metrics at the same time.
- Example experiments demonstrate that statistical designs can be used to optimize the parameters introduced to FSSW including rotating speed, dwell time, and pin diameter to improve the mechanical and heat properties of PA6 welds [55].

### 9.2. Machine Learning and Data-Driven Optimization Approaches

**Figure 13:**
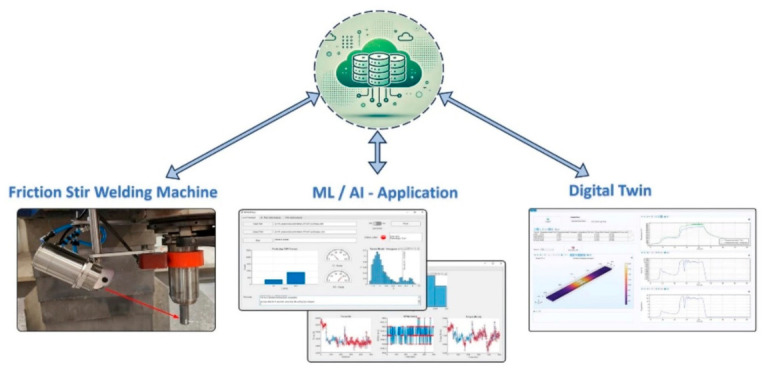
Schematic illustration of machine learning and digital twin-based modeling approaches for monitoring and control of friction stir welding [57].

Traditional research has also begun to move to the concept of machine learning (ML) and general artificial intelligence (AI) to pre-empt joint quality and optimize process parameters, which tend to be more accurate in prediction and less physical experiments (see Figure 13). Artificial Neural Networks (ANN), Support vector machines (SVM), random forests, and hybrid-based machine learning (ANN + PSO, ANFIS + GA) have been used in friction stir welding processes and exhibit superior consistency since they can be used to predict the effects of mechanical property, and the impact of parameters [56].

ML approaches also enable

**Figure 14:**
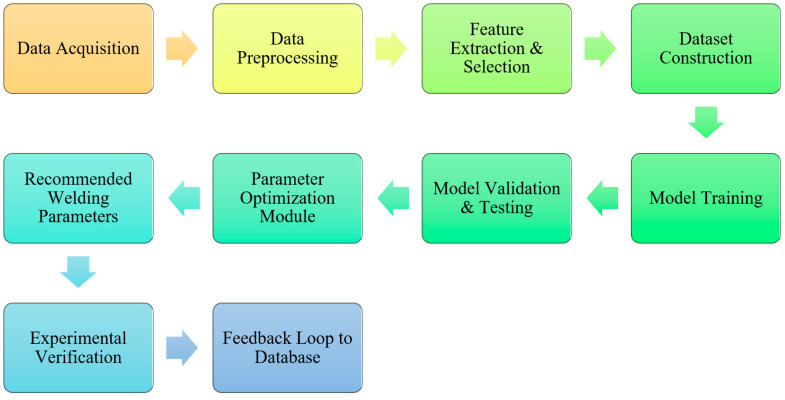
Workflow of an ML-assisted welding optimization system.

- Multi-response outputs (e.g., tensile strength, hardness) caused by process conditions can be predicted, and there is no need to rely on empirical models (See Figure 14).
- Estimating latent relationships in variables when it is hard to do so in classical regression.
- Combination with optimization algorithms (PSO, TLBO, genetic algorithms, etc.) to find Pareto-optimal sets of parameters.

Emerging Trends:

- ML + digital twin (DT) systems of real-time predictive control of the parameters of welding, allowing adaptive control of processes to avoid flaws and enhance the stability of the process [57].
- Apply an ensemble model (e.g., Random Forest) to trade off interpretability and predictive accuracy in joint quality prediction [58].

### 9.3. Real-Time Monitoring, Process Control, and Energy-Efficient Welding Strategies

Monitoring in real time and adaptive process regulation has become very important in enhancing the quality of the welds, reliability, and sustainability of USW and FSSW of polymers and metal–polymer hybrids. The new welding sites in contrast to traditional open-loop systems incorporate the concept of multi- sensor architecture which constantly measures the process variables like axial force, torque, the plunge depth, temperature, vibration amplitude, and acoustic emission. These measurements immediately give information about the economy of the interaction of tools and workpieces, the heat generation, the behavior of material flow, and the interfacial bonding process. Such data can then be analyzed in real time to identify instabilities that present defect precursors; i.e., torque oscillations instead of adequate plasticization or abnormal temperature spikes instead of over-heating can be identified early enough to take corrective measures before joint degradation takes place [59].

The latest research indicates that it is not only possible but also advisable to use intelligent monitoring systems integrating sensor-based acquisition and machine learning-based classification. As an example, the WeldMon tool condition monitoring system, USW, was able to classify with high accuracy (~95) on the signal feature extractor using pattern recognitions algorithms [60]. Equally, IIoT-based friction stir welding instruments combining temperature and force sensors with neural network and fuzzy logic instruments have demonstrated to improve the predictive capability of the system and adaptive control output. This is one of several developments indicating a larger movement toward data-centric, cyber–physical welding systems, which will be in line with the Industry 4.0 and Industry 5.0 manufacturing models. The main elements of the real-time monitoring systems include:

- Sensors Unusual Thermocouples, infrared sensors, load cells, torque transducers, accelerometers, and acoustic emission sensors detect dynamic thermal and mechanical data.
- Signals: Interpretability is improved by signal processing: Filters (FFT, wavelet transform), feature (RMS torque, peak force, energy index) and statistical normalization.
- Media feedback Closed-loop controllers can dynamically control the rotational speed, the plunge depth, dwell time, or the ultrasonic amplitude to achieve stability.
- Condition Monitoring: Predictive analytics will help to detect defects early and perform preventive maintenance, which lowers scrap and downtime [61].

The microstructural evolution and weld quality are connected to energy efficiency inseparableness. In USW and FSSW, polymer softening, mixing the materials, diffusing interfaces, as well as possible intermetallic layers are controlled by energy input. Over energy causes thermal degradation, the formation of voids, and brittle interfaces, and under energy causes lack of bonding. Therefore, optimization involves some trade-off between mechanical work and heat production within a specific thermo-mechanical processing range. Reduction in energy consumption can be achieved by optimization methods that limit dwell time, axial force, or ultrasonic amplitude and provides enough interfacial bonding temperature [62]. Real-time adaptive control helps directly in terms of sustainability because it helps in eliminating unnecessary high-energy operation after bonding requirements are met. This helps to decrease thermal overstressing and improve micro-structural uniformity. Furthermore, performance in AI-assisted systems and digital twin frameworks allows simulating the virtual way of processes and predicting the parameters and reducing the necessity of the massive experiments. Welding systems based on digital twins that combine sensor data on a real-time basis with predictive models have shown an increased joint strength, a decrease in process variability and an overall energy need [63].

## 10. Applications and Industrial Implementation

### 10.1. Automotive Lightweight Structures

The automotive industry has aggressively adopted USW and FSSW to meet toughness, lightweight, and fuel efficiency targets. Metal–polymer joints, particularly aluminum–thermoplastic and composite laminates, provide superior specific strength and crash energy absorption compared to traditional steel assemblies. Solid-state joining techniques such as FSSW preserve base material integrity while minimizing heat-affected zones and distortion, making them suitable for multi-material automotive architectures. Table 18 shows the automotive applications of USW/FSSW [64,65,66,67].

- FSSW Aluminum–Polymer Joints: FSSW bonds aluminum (e.g., AA6061) to polymers like PP using interlayers to promote interfacial reactions, yielding strong lap joints for panels and battery enclosures without keyholes. Mazda’s automotive applications demonstrate defect-free joins, with optimized parameters (600 rpm, 3 s dwell) achieving high shear strength in thin sheets [64]. FSSW joins Al to CFRP for bumper beams, reducing distortion versus adhesives [65].
- USW Aluminum–Polymer Joints: USW creates reliable metal–polymer hybrids via vibrational energy and minimal heat, ideal for thin dissimilar sheets (<1 mm) in hybrid vehicle components like door modules. It forms refined grain structures with low intermetallic, supporting 20–30% weight savings in multi-material bodies [66]. USW enables polymer–Al battery tabs in hybrids, verified by SEM for uniform interfaces [67].

### 10.2. Aerospace and Transportation Systems

In aerospace structures, minimizing weight while maintaining fatigue resistance and structural integrity is critical. Solid-state processes like USW and FSSW are advantageous because they avoid melting-related intermetallic brittleness, which is common in fusion welding. Table 19 shows the aerospace and transportation systems applications of USW/FSSW [67,68,69].

- Aerospace Aluminum–Polymer Structures: FSSW joins aluminum alloys to polymers or CFRP for fuselage skins and wing panels, minimizing distortion versus riveting; Boeing reports near-zero defects in high-strength Al structures. USW suits thin Al–polymer laminates in satellites and aircraft interiors, providing low-heat bonds with refined microstructures [68].
- Transportation Systems: In high-speed trains like Hitachi’s Class 395 “Javelin,” FSSW creates lightweight aluminum extrusion bodies with polymer composites, enhancing fatigue resistance [69]. USW bonds Al–polymer for bus and rail panels, achieving cycle times under 1 s for efficient assembly [67].

### 10.3. Electronics, Medical, and Consumer Products

FSSW and USW are used to form aluminum–polymer joints in electronics, medical devices, and consumer products, providing low-heat, high-speed bonding of small, lightweight assemblies. Table 20 shows the electronics, medical, and consumer products applications of USW/FSSW [1,70].

- Electronics Applications: USW welds aluminum sheets onto polymer substrates in battery tabs, wire harnesses and busbar with great shear strength (up to 84.8 MPa with interlayers) and electrical conductivity of less than 0.5–1 s cycles. It is superior when it comes to electronics packaging in AA6061 where it involves reducing defects through interfacial plastic deformation and acoustic softening [70].
- Medical Devices: USW: hermetically sealed aluminum–polymer implant and disposable enclosures are sealed by refined micro structures which ensure sterility and biocompatibility; energy (0.6–1.5 kJ) avoids thermal damage. FSSW facilitates the catheter assembly and housings and offers unrestricted thin foil laps [1].

## 11. Emerging Trends and Future Research Directions

As solid-state joining methods such as USW and FSSW continue to mature, new research directions have begun to shape the evolution of polymer and hybrid joining technology. These trends span hybrid joining strategies, improved tool designs, sustainability through recycled and bio-based materials, digital twin and physics-informed models, and the need for standardization in testing and data reporting [66].

### 11.1. Key Emerging Research Directions

- Hybrid Joining Techniques: Hybrid joining approaches that combine USW, FSSW, and related processes are gaining importance as a means to overcome individual process limitations. While USW enables rapid heating, it is constrained by geometry and thickness, whereas FSSW offers stronger joints but faces challenges in metal–polymer joints. Their integration allows simultaneous use of vibrational energy and mechanical stirring, improving interfacial bonding and material compatibility [71]. Polymer stir welding (PSW) has also shown promise by enabling controlled polymer melting and mechanical interlocking with metallic substrates [72]. Future work should focus on systematic performance evaluation under mechanical, thermal, and environmental loading.
- Advanced Tool and Horn Designs: Tool and horn design play a critical role in controlling heat generation, material flow, and joint quality. Additive manufacturing has enabled topology-optimized tools that improve material mixing, while adaptive horn designs enhance vibration distribution and reduce stress concentrations. Surface-engineered tools, including textured and coated interfaces, further improve bonding efficiency [66]. However, integrating material-specific behavior into tool design and validating performance under realistic conditions remain key research needs.
- Joining of Recycled and Bio-Based Polymers: The shift toward sustainable manufacturing has increased interest in recycled and bio-based polymers. These materials pose challenges due to variability in properties, degradation, and lower thermal stability. Research is still limited, particularly for hybrid systems. Future studies should focus on welding recycled polymers such as PA, PET, and PLA, optimizing process parameters to minimize degradation, and evaluating performance under service conditions. Incorporating life-cycle assessment will be essential to quantify environmental benefits.
- Digital Twins and Physics-Informed Modeling: Digital twin technology and physics-informed models are emerging as powerful tools for process optimization and predictive control. By integrating thermal, mechanical, and viscoelastic models with real-time sensor data, digital twins can predict temperature evolution, deformation, and defect formation with high accuracy [73]. Recent studies demonstrate the integration of data-driven models with numerical simulations to accurately capture temperature distributions and enable real-time optimization of process parameters, leading to improved joint quality and efficiency. Advanced model architectures have shown enhanced predictive performance, particularly under complex thermal conditions [74]. These approaches reduce experimental effort and enable adaptive control. However, challenges such as dependence on high-quality datasets, process variability, and computational cost remain, necessitating further development of robust and scalable frameworks. Future work should focus on multi-physics modeling of polymer–metal systems and integration with machine learning for enhanced prediction and optimization.

### 11.2. Gaps in Standardization and Testing Protocols

Despite rapid advancements in USW and FSSW for polymer and metal–polymer hybrid systems, a major limitation remains the lack of standardized testing protocols. Variations in specimen geometry, joint configuration, loading conditions, and environmental exposure make cross-study comparisons difficult, hindering benchmarking, qualification, and industrial adoption. This issue is more pronounced in hybrid joints due to differences in thermal expansion, viscoelastic behavior of polymers, and complex interfacial interactions, which are not adequately addressed by conventional metallic or adhesive joint standards. Major gaps include the absence of unified mechanical and environmental test matrices, limited fatigue, and long-term durability data under realistic service conditions, and insufficient studies on environmental degradation such as thermal cycling, moisture ingress, and chemical exposure. Inconsistent reporting of process parameters (e.g., energy input, dwell time, rotational speed) and the lack of normalized performance metrics further restrict data comparability. Additionally, non-standardized failure mode classification limits mechanistic understanding of joint behavior. Fatigue and durability performance remain particularly underexplored, especially considering the time-dependent and temperature-sensitive nature of polymers. To address these challenges, a standardized test suite is proposed (Table 21), covering key mechanical and environmental evaluations.

A structured environmental aging framework is also essential, including thermal cycling (−40 °C to 120 °C), high-humidity exposure (85% RH at 85 °C), chemical resistance testing, and UV exposure to simulate real service conditions. Standardized testing protocols would enable consistent benchmarking, accelerate certification, and generate high-quality datasets for predictive modeling and machine learning applications. While advances in hybrid joining, tool design, sustainable materials, and digital optimization are emerging, the absence of unified qualification frameworks continues to limit the transition from laboratory-scale studies to large-scale industrial implementation.

## 12. Conclusions

Recent research on joining polymers and metal–polymer hybrid structures using USW and FSSW has expanded rapidly in response to the demand for lightweight, high-performance assemblies in aerospace, automotive, and consumer applications. Both processes are solid-state in nature and rely on localized heat generation through high-frequency vibrational energy in USW and frictional heating coupled with plastic deformation in FSSW, thereby minimizing thermal degradation and residual stresses associated with conventional fusion welding. In USW, joint formation is governed primarily by viscoelastic dissipation, interfacial friction, and mechanical interlocking, particularly critical for polymer–metal hybrids where chemical bonding is inherently limited. These mechanisms are strongly enhanced by surface preparation techniques such as laser texturing and mechanical grooving, which increase effective contact area and promote robust interfacial anchoring.

By comparison, FSSW is characterized by intense dynamic recrystallization, intense plastic deformation and movement of material through the stir zone, resulting in microstructural refining and enhancement of load transfer among different materials. Microstructural studies agree that incessantly dynamic recrystallization results in such fine equiaxed grains in metallic areas and that the existence of defects including, voids, pores, within-target metallic pieces makes a final impact in counting joint integrity. On the basis of performance perspective, the joint quality of USW with entirely polymer materials is strongly associated with interfacial morphology, as textured metal surfaces enhance meso-mechanical interlocking and change failure modes involving adhesive to mixed cohesive-adhesive fracture modes, enhancing joint strength and reliability. FSSW joints in their turn are highly responsive to such parameters of the process as rotating speed, depth of the plunge, and dwell time, and optimal parameters result in highly significant increases in lap shear strength and hardness, but difficulties in defect suppression and interface uniformity are still present.

Although this has been advanced, there are a number of research gaps. To advance past empirical parameter-performance correlations, future work ought to target the physics-based interpretations of process monitoring in situ and post-weld microstructural and mechanical analyses to formidable mechanism-performance associations. Methodical research on surface engineering, including scale, topology, and chemistry, is still scarce, and ought to be reinforced by quantitative characterization of surfaces, related to joint strength and fatigue pattern. In addition, the performance of fatigue, durability, and life-cycle behavior at the loading conditions of service relevance are yet to be studied, especially in safety-critical automotive and aerospace use cases. Improvement of multiphysics numerical coding to also coupler viscoelastic heating, thermo-mechanical flow, and interfacial bonding criteria of both USW and FSSW are also in need. Last but not least, due to the absence of standardized joint geometries, testing regimes, and reporting measures, it remains a significant barrier to useful comparisons among studies, and benchmarking frameworks are necessary to create an accelerated adoption of such advanced joining technologies by industries.

## Figures and Tables

**Table 1:** Comparison of conventional joining methods and their limitations for metal–polymer joints.

Joining Method	Principle	Typical Applications	Key Advantages	Major Limitations for Metal-Polymer Joints
Mechanical Fastening (Bolts, Screws, Rivets)	Mechanical interlocking through drilled holes	Automotive panels, consumer products	Simple, reversible, no thermal damage	Stress concentration, added weight, hole-induced damage in polymers, poor fatigue and sealing performance
Adhesive Bonding	Chemical adhesion between metal and polymer surfaces	Aerospace interiors, lightweight structures	Uniform stress distribution, good surface finish	Surface preparation sensitive, long curing time, degradation under heat/moisture, limited high-temperature strength
Resistance Spot Welding (RSW)	Joule heating through electrical resistance	Metal–metal joints in automotive	High productivity for metals	Not suitable for polymers; polymer degradation and lack of electrical conductivity
Laser Welding/Laser Assisted Joining	Localized heating using laser energy	Precision polymer–polymer or metal–polymer joints	Non-contact, precise energy input	High equipment cost, narrow process window, thermal degradation of polymers, limited joint thickness
Hot Plate Welding	External heated plate melts joining surfaces	Thermoplastic components	Strong polymer–polymer joints	Not applicable to metals; excessive heat input causes polymer distortion
USW (Conventional)	High-frequency vibration generates frictional heat	Thermoplastics, thin metal foils	Fast cycle time, low energy consumption	Limited penetration depth, difficulty joining thick metals, tooling wear
Friction Welding (Conventional Rotary/Linear)	Frictional heat under axial pressure	Metal–metal joints	Solid-state bonding, strong joints	Poor compatibility with polymers due to melting, degradation, and flow instability
Brazing/Soldering	Filler material melts to bond substrates	Electronics, metal assemblies	Good gap filling	Polymer thermal degradation, poor wetting, environmental concerns
Arc Welding (MIG/TIG)	Fusion welding using electric arc	Structural metal joints	High strength, mature technology	Completely unsuitable for polymers; excessive heat input and thermal mismatch

**Table 2:** Key thermoplastics and polymer composites used in USW and FSSW for metal–polymer hybrid joints.

Material	Typical Applications	Key Benefits for USW/FSSW	Primary Challenges in Hybrid Joining
Polypropylene (PP)	Automotive interior panels, battery casings, lightweight enclosures	Low density, good flow behavior, low processing temperature, cost-effective	Limited high-temperature performance, low stiffness, weak interfacial bonding with metals
Polyamide (PA6/PA66)	Structural automotive components, clips, brackets	High strength, good thermal stability, excellent weldability, strong adhesion to metals	Moisture sensitivity, degradation at high heat input
Glass Fiber Reinforced Polyamide (GF-PA)	Load-bearing automotive and aerospace parts	Improved stiffness and strength, good fatigue resistance	Fiber breakage during welding, anisotropy, reduced interfacial flow
Carbon Fiber Reinforced Polyamide (CF-PA)	Lightweight structural and semi-structural components	High specific strength and stiffness, enhanced fatigue resistance	Poor through-thickness bonding, fiber damage, limited polymer flow
Acrylonitrile Butadiene Styrene (ABS)	Consumer products, automotive trims, housings	Good impact resistance, stable processing window	Lower thermal resistance, limited performance in high-load joints
Polyetheretherketone (PEEK)	Aerospace structures, biomedical implants, high-temperature components	Exceptional thermal stability, chemical resistance, high mechanical strength	High processing temperature, narrow welding window, cost
Polyetherimide (PEI)	Aerospace interiors, electrical housings	High T_g_, dimensional stability	Brittleness, sensitivity to process parameters
Polycarbonate (PC)	Transparent structural parts, electronic housings	High impact strength, good energy absorption in USW	Susceptible to thermal degradation, residual stresses
Thermoplastic Polyurethane (TPU)	Flexible joints, damping layers, seals	High elasticity, energy dissipation, good interfacial conformity	Low stiffness, limited load-bearing capability
Polypropylene-based Composites (PP-GF/PP-CF)	Automotive panels, structural inserts	Enhanced stiffness with low weight	Fiber–matrix debonding, reduced weld quality at high fiber content

**Table 3:** Key metallic materials used in FSSW/USW for hybrid joints.

Metallic Material	Typical Applications	Key Advantages for Hybrid Joining	Challenges in USW/FSSW with Polymers
Aluminum Alloys (AA5xxx, AA6xxx, AA7xxx)	Automotive body panels, aerospace structures, battery enclosures	Lightweight, good thermal conductivity, high compatibility with USW and FSSW	Oxide layer formation, interfacial debonding, thermal softening
Magnesium Alloys (AZ31, AZ91)	Lightweight automotive components, electronics housings	Very low density, excellent weight reduction potential	Oxidation, low corrosion resistance, narrow processing window
Low-Carbon Steel (IF steel, mild steel)	Automotive frames, reinforcement inserts	High strength, low cost, structural reliability	High stiffness mismatch with polymers, increased tool wear
Advanced High-Strength Steels (AHSS)	Crash-resistant automotive structures	High load-bearing capability, good fatigue resistance	High heat generation, tool degradation, limited plastic flow
Stainless Steels (AISI 304, 316)	Medical devices, aerospace fittings	Corrosion resistance, durability	High hardness, increased energy demand, interface cracking
Titanium Alloys (Ti-6Al-4V)	Aerospace structures, biomedical implants	High specific strength, excellent corrosion resistance	Poor thermal conductivity, high process temperature requirement
Copper and Copper Alloys (Cu, Cu-Cr-Zr)	Electrical connectors, battery tabs	Excellent electrical and thermal conductivity	Excessive heat dissipation, polymer degradation risk
Zinc-Coated Steels (GI, GA steels)	Automotive panels, corrosion-resistant parts	Improved corrosion resistance, good USW compatibility	Coating degradation, zinc vaporization at high energy input
Aluminum Matrix Composites (AMCs)	Aerospace and high-performance structures	High stiffness-to-weight ratio	Abrasive reinforcement, tool wear, limited interfacial mixing
Surface-Treated Metals (anodized Al, laser-textured steel)	Hybrid joints with enhanced bonding	Improved mechanical interlocking and adhesion	Added processing complexity, surface damage during welding

**Table 4:** Representative polymer–metal pairs in the FSSW/USW literature.

Polymer	Metal	Joining Technique	Notable Outcome
PA6	AA6082	FSSW	Enhanced mechanical interlocking with textured metal surfaces leading to improved joint strength through meso-mechanical interlocking mechanisms [11].
PMMA	Aluminum (AA6061)	FSW/FSSW	Joints achieved significant tensile strengths; PMMA fusion and mixing behavior influences final joint quality [1].
PA66	AA7075	Friction-Assisted Joining	Numerical and experimental work showed temperature and force distributions affect joint integrity [12].
GF/PA6	AA6082-T6	FSSW	Different surface pretreatments (grinding vs. groove) led to distinct micro-mechanical vs. meso-mechanical interlocking, improving joint strength [13].
PE	Aluminum alloys (AA6061)	FSW	Achieved hybrid joint formation with measurable tensile strength; parameter optimization is critical [1].
PC	AA6061	FSW	Hybrid joints with measurable mechanical performance, albeit lower than base polymer due to dissimilarity challenges [1].
PA (various)	Steel	FSSW	Suggests process parameter sensitivity and challenge of defect-free hybrid joining (extension of dissimilar welding studies) [14].

**Table 5:** Heat generation mechanisms in the USW of thermoplastics.

Heat Generation Mechanism	Physical Origin	Dominant Welding Stage	Governing Parameters	Heat Generation Characteristics	Effect on Joint Formation
Interfacial (surface) friction	Relative oscillatory motion between microscopic surface asperities at the polymer–polymer interface	Initial vibration stage (before softening)	Surface roughness, contact pressure, vibration amplitude	Localized heating concentrated at asperity contacts; rapid temperature rise at interface	Initiates polymer softening and promotes intimate contact; critical for triggering melt formation
Viscoelastic dissipation (bulk heating)	Internal molecular friction due to time-dependent viscoelastic response under cyclic loading	Dominant after temperature approaches T_g_	Frequency (ω), vibration amplitude (ε), loss modulus (E″), polymer rheology	Distributed subsurface heating; volumetric energy dissipation proportional to ω·E″·ε^2^	Governs melt growth, flow behavior, and weld consolidation; controls final joint strength
Combined friction–viscoelastic heating	Transition from asperity friction to bulk viscoelastic energy dissipation	Intermediate stage during weld development	Temperature, pressure, amplitude, polymer thermal properties	Shift from surface-localized to bulk-dominated heat generation	Ensures stable molten layer and uniform interfacial bonding

**Table 6:** Influence of key USW parameters on heat generation and joint performance.

Process Parameter	Typical Range	Primary Role in Welding	Effect on Heat Generation	Influence on Joint Quality	Defect Risks at Extreme Values
Vibration amplitude	~10–100 µm	Controls cyclic strain and energy input at the interface	Higher amplitude increases viscoelastic dissipation and melting rate	Enhances interfacial fusion and joint strength at optimal levels	Excessive flash, non-uniform melting, polymer degradation, fiber breakage in composites
Frequency	20, 30, 40 kHz (resonant)	Governs vibration rate and penetration depth of energy	Higher frequency increases heating rate but reduces penetration depth	Affects melt distribution and material response, especially for thin or stiff polymers	Limited flexibility due to system resonance; mismatch can reduce energy transfer efficiency
Welding pressure (clamping force)	Material- and geometry-dependent	Ensures intimate contact and controls molten layer thickness	Promotes frictional heating and stabilizes viscoelastic dissipation	Improves consolidation, reduces voids, and enhances joint integrity	Low pressure: weak bonding; high pressure: melt expulsion, reduced bond area

**Table 7:** Influence of energy director geometry on interfacial morphology and weld performance.

Energy Director Geometry	Heat Concentration Efficiency	Collapse Behavior	Typical Applications	Advantages	Limitations
Triangular	Very high	Rapid, controlled collapse	General thermoplastics, composites	Fast weld initiation, uniform melting, high joint strength	Sensitive to amplitude; risk of flash if over-energized
Semicircular	Moderate	Gradual collapse	Thick sections, stiff polymers	Improved process stability, reduced flash	Slower melting, slightly lower peak strength
Rectangular	Low–moderate	Progressive flattening	Large weld areas, rigid parts	Robust geometry, easy molding	Less efficient energy focusing
Textured metal surface (hybrid)	High (localized)	Polymer penetration into texture	Polymer–metal joints	Strong mechanical interlocking, enhanced load transfer	Requires precise surface preparation

**Table 8:** FSSW process parameters and their influence on joint strength and performance.

Parameter	Primary Influence	Typical Effect on Joint Performance
Shoulder diameter	Heat generation, plasticized volume	Larger weld area and improved joint strength
Pin profile	Material flow patterns	Enhanced material mixing and interfacial bonding
Plunge depth	Stirred zone size	Moderate depth improves strength; excessive depth may cause defects
Dwell time	Thermal exposure duration	Increased dwell enhances consolidation but may degrade polymers

**Table 9:** Key differences between FSSW of metals and polymers.

Aspect	FSSW of Metals	FSSW of Polymers
Thermal conductivity	High thermal conductivity leads to rapid heat diffusion and relatively broad softened zones	Low thermal diffusivity causes localized heat retention and steep thermal gradients
Dominant softening mechanism	Plastic deformation governed by dislocation motion and dynamic recrystallization	Viscoelastic and viscoplastic chain mobility activated once temperature exceeds T_g_
Material flow behavior	Stable plastic flow with continuous stirring and material consolidation	Highly temperature-dependent flow; excessive heat can cause degradation or void formation
Stir zone characteristics	Fine equiaxed grains formed due to dynamic recrystallization	Distinct stir zones characterized by molecular interdiffusion and chain entanglement
Heat-affected region	Clearly defined TMAZ and heat-affected zone (HAZ)	Narrow or indistinct HAZ; properties governed mainly by T_g_-related transitions
Defect tendencies	Hook defects, kissing bonds, and voids due to insufficient material flow	Voids, thermal degradation, and incomplete bonding due to localized overheating
Tool design sensitivity	Tool geometry primarily influences material flow and grain refinement	Tool geometry critically controls heat localization and polymer flow stability
Process window	Relatively wider and more forgiving	Narrower window requiring precise control of temperature and dwell time
Joint strength control	Governed by grain refinement, nugget size, and metallurgical bonding	Governed by interfacial diffusion, molecular entanglement, and thermal stability

**Table 10:** Interfacial bonding mechanisms.

Hybrid Type	Dominant Mechanism	Evidence/Effect
Polymer–Metal (smooth)	Limited mechanical interlocking	Lower joint strength
Polymer–Metal (textured/grooved)	Enhanced mechanical anchoring	Higher shear strength
Polymer–Metal with surface treatments	Combined mechanical + adhesive	Improved performance

**Table 11:** Comparison of USW and FSSW process mechanics.

Feature	USW	FSSW
Heat Generation	Viscoelastic and frictional at interface	Frictional heat from rotating tool
Material Flow	Localized melt and solidification at interface	Bulk stirring and plastic flow
Bonding Mechanism	Melt diffusion + pressure fusion	Mechanical interlocking + plasticized flow
Joint Geometry	Spot weld with minimal disturbance	Stirred zone with nugget and thermo-mechanical region
Control Parameters	Frequency, amplitude, pressure, time	Rotational speed, plunge depth, dwell time, tool design

**Table 12:** Influence of polymer chain orientation and crystallization on joint performance in polymer welding processes.

Aspect	USW/FSSW	Effect on Joint Performance	Characterization Techniques
Chain mobility	High-frequency ultrasonic vibrations promote localized molecular mobility at the weld interface	Enhances interdiffusion and chain entanglement across the interface, increasing joint strength	Indirectly inferred from DSC and birefringence analysis
Chain orientation	Oscillatory shear and compressive stresses induce partial chain alignment near the bonded zone	Improves load transfer efficiency and reduces interfacial failure	TEM, birefringence imaging
Interfacial entanglement	Elevated temperature and vibration time enable polymer chains to interpenetrate and entangle	Primary mechanism governing strength in polymer–polymer welds	Fractography (SEM), mechanical testing
Crystallization behavior	Partial melting followed by controlled cooling leads to recrystallization in semi-crystalline polymers	Refines microstructure, enhances stiffness, and reduces mechanical anisotropy	DSC, XRD
Microstructural refinement	Reorganization of crystalline and amorphous phases occurs under combined thermal and mechanical action	Improves joint durability and resistance to creep or fatigue	XRD, SEM, TEM

**Table 13:** Interfacial features and their influence on metal–polymer joint performance.

Interface Feature	Dominant Mechanism	Effect on Joint Performance
Smooth interface	Limited interlocking	Low strength, adhesive failure
Rough/textured interface	Polymer penetration into asperities	Higher strength, improved load transfer
Embedded metal fragments	Mixed mechanical anchoring	Enhanced stiffness and toughness
Voids/kissing bonds	Poor consolidation	Reduced fatigue life and reliability

**Table 14:** Comparative mechanical performance of hybrid joints.

Study/Joint Type	Materials	Welding Type	Static Strength (MPa)	Durability/Retention (%)	Fatigue Performance	Key Finding
Seal-flow FSLW	Al + GF-PEEK	FSLW	~26 MPa (lap shear)	—	—	Improved strength via vortex flow and interlocking [40]
Anodized Al/Composite	Al + CFRP	FSSW	~28–32 MPa (initial lap shear)	~80% strength retention after environmental exposure (humidity/aging)	—	Good adhesion reduces humidity ingress [41]
Friction spot joints	Al2024 + CFRP	FSpJ	~30 MPa (static lap shear)	—	~30–35% of static strength (fatigue limit)	S-N behavior indicates endurance at low stress levels [42]

**Table 15:** Fatigue and impact performance of polymer and metal–polymer joints.

Aspect	Loading/Observation	Key Outcome	Implication for Joint Design
Fatigue endurance	Cyclic loading (R = 0.1, ~5 Hz)	Sustains up to 10,610^6106^ cycles at 25–35% of static strength	Indicates stable interfaces and effective load transfer
Residual strength	Post-fatigue quasi-static testing	Minimal strength degradation	Confirms durability of mechanically interlocked joints
Crack morphology	SEM fractography	Striations in resin-rich regions, mixed failure	Polymer matrix and interface both govern fatigue damage
Impact/dynamic response	Limited experimental data	Strong dependence on interface quality	Critical need for dynamic and impact-focused studies

**Table 16:** Failure modes and environmental effects in polymer and hybrid welded joints.

Aspect	Observed Behavior	Effect on Joint Performance
Adhesive failure	Weak interface, moisture ingress	Low strength, poor durability
Cohesive/mixed failure	Strong interlocking and bonding	Higher strength, stable load transfer
Excessive heat input	Polymer degradation, flash	Strength reduction, defects
Environmental aging	Humidity-induced interface weakening	20–30% strength loss without protection

**Table 17:** Constitutive models commonly used in welding simulations.

Material System	Typical Constitutive Model	Key Features
Metallic alloys	Johnson–Cook (J–C) model	Captures strain hardening, strain-rate sensitivity, and thermal softening under large plastic deformation
Polymers	Viscoelastic/Viscoplastic models (Maxwell, Prony series, Perzyna)	Describes time-dependent deformation, temperature sensitivity (near T_g_), and rate-dependent plastic flow
Metal–polymer joints	Multi-phase/Layered constitutive models	Represents distinct thermo-mechanical behavior of metal and polymer phases with interfacial coupling effects

**Table 18:** Automotive applications of USW/FSSW.

Component	Process	Joint Strength	Cycle Time	Key Benefit
Battery tabs	USW	High shear (>750 J)	<1 s	Low heat, uniform interface
Hybrid body panels	FSSW	70–80% base metal	2–5 s	Minimal distortion, no keyhole
Door modules	USW	Refined grains, low IMC	<1 s	Vibration bonding for thin sheets
Bumper beams	FSSW	Interlayer-enhanced	3–5 s	Al-CFRP hybrid, reduced weight
Crash reinforcements	FSSW	Improved fatigue life	3–6 s	Defect-free microstructure

**Table 19:** Aerospace and transportation systems applications of USW/FSSW.

Component	Process	Joint Strength	Cycle Time	Key Benefit
Fuselage skins	FSSW	70–90% base metal	2–5 s	Weight savings vs. rivets
Wing panels	USW	High shear, low IMC	<1 s	No porosity in hybrids
Train extrusions	FSSW	Improved fatigue	3–6 s	Distortion-free Al-polymer
Satellite frames	USW	Refined grains	<1 s	Vibration-resistant bonds

**Table 20:** Electronics, medical, and consumer products applications of USW/FSSW.

Component	Process	Joint Strength	Cycle Time	Key Benefit
Battery packs	USW	84 MPa peak shear	<0.8 s	High conductivity, low IMC
Device housings	FSSW	70% base metal	2–3 s	No porosity in Al-polymer
Wearables	USW	Refined grains	<0.5 s	Compact, vibration bonds
Packaging seals	USW	Enhanced interface	<1 s	Clean, energy-efficient

**Table 21:** Proposed standardized test suite for hybrid USW/FSSW joints.

Test Type	Purpose	Justification for Hybrid Joints	Key Parameters to Standardize
Lap Shear Test	Static strength evaluation	Primary load mode in automotive and aerospace lap joints	Overlap ratio, weld area normalization
Peel Test	Interfacial adhesion strength	Evaluates polymer–metal bonding integrity	Peel angle, loading rate
Cross-Tension Test	Out-of-plane performance	Simulates crash or impact-induced loading	Nugget diameter definition
Fatigue Test (High/Low Cycle)	Durability assessment	Captures cyclic degradation and interfacial fatigue	Stress ratio (R), frequency
Impact Test	Energy absorption	Relevant for crashworthiness	Impact velocity, striker geometry
Thermal Cycling	Thermal stability	Simulates service in automotive/aerospace	Temperature range, cycle number
Moisture/Environmental Aging	Chemical stability	Polymers sensitive to humidity and chemicals	Exposure duration, humidity %
Creep Test	Time-dependent deformation	Polymers exhibit viscoelastic creep	Load level, temperature

## Data Availability

No new data were created or analyzed in this study. Data sharing is not applicable to this article.
